# Investigation of Rheological, Mechanical, and Viscoelastic Properties of Silica-Filled SSBR and BR Model Compounds

**DOI:** 10.3390/polym16223212

**Published:** 2024-11-19

**Authors:** Anmol Aggarwal, Nico Hackel, Fabian Grunert, Sybill Ilisch, Mario Beiner, Anke Blume

**Affiliations:** 1 Department of Elastomer Technology and Engineering, University of Twente, Drienerlolaan 5, 7522 NB Enschede, The Netherlands; a.aggarwal@utwente.nl (A.A.); a.blume@utwente.nl (A.B.); 2 IMWS Institute for Microstructure of Materials and Systems, Walter-Hülse-Straße 1, 06120 Halle (Saale), Germany; 3 Faculty for Natural Sciences II, Martin-Luther-University Halle-Wittenberg, Heinrich-Damerow-Str. 3, 06120 Halle (Saale), Germany; 4 Synthos Schkopau GmbH, Str. E 17, 06258 Schkopau, Germany; sybill.ilisch@synthosgroup.com

**Keywords:** SSBR, BR, functionalized polymers, silica, silane, mechanical and dynamic properties, viscoelasticity

## Abstract

Active fillers such as carbon black and silica are added to rubber to improve its mechanical and viscoelastic properties. These fillers cause reinforcement in rubber compounds through physical and/or chemical interactions. Consequently, the compounds’ rheological, mechanical, and viscoelastic behavior are affected. Changing the filler loading influences these properties due to the different interactions (filler-filler and filler-polymer) taking place in the compounds. In addition, rubbers with varying microstructures can interact differently with fillers, and the presence of polymer functionalization to enhance interactions with fillers can further add to the complexity of the network. In this work, the effects of different loadings (0–108 phr/0–25 vol. %) of a highly dispersible grade of silica with three types of solution styrene-butadiene rubbers (SSBR) and one butadiene rubber (BR) on their rheological, mechanical, and viscoelastic properties were investigated. It was observed that the Mooney viscosity and hardness of the compounds increased with an increasing filler loading due to the increasing stiffness of the compounds. Payne effect measurements on uncured compounds provided information about the breakdown of the filler-filler network and the extent of the percolation threshold (15–17.5 vol. %) in all the compounds. At high filler loadings, the properties for BR compounds worsened as compared to SSBR compounds due to weak polymer-filler interaction (strong filler-filler interaction and the lower compatibility of BR with silica). The quasi-static mechanical properties increased with the filler loading and then decreased, thus indicating an optimum filler loading. In strain sweeps on cured rubber compounds by dynamic shear measurements, it was observed that the type of rubber, the filler loading, and the temperature had significant influences on the number of glassy rubber bridges in the filler network and, thus, a consequential effect on the load-bearing capacity and energy dissipation of the rubber compounds.

## 1. Introduction

Unfilled rubber, in particular, synthetic rubber, has relatively poor mechanical properties. The addition of active fillers to rubber is necessary for achieving further improvements in these mechanical properties [1]. Silica is a well-established reinforcing filler in rubber compounds. Its high specific surface area and ability to link chemically with the polymer, especially in the presence of silane, provide an ideal platform for interaction with the polymer matrix [2,3]. Together with the addition of silane coupling agents, the compatibility between silica surfaces and rubber matrices is enhanced, thus creating a robust reinforcing network [4,5]. Consequently, the silica-silane system significantly contributes to the enhancement of the mechanical and viscoelastic properties of rubber compounds as compared to conventional filler systems such as carbon black [6,7].

Polymer properties, such as molecular weight, microstructure, viscosity, etc., affect the processability, rheological and mechanical properties, and energy dissipation behavior of rubber compounds [8]. As its molecular weight is increased, the total number of free chain ends in a rubber sample is reduced and the energy dissipation of the cured compound is commonly expected to decrease [9]. However, the processability of compounds becomes compromised, because this increased molecular weight results in a high viscosity of the compound, leading to a poor flowability and high shear forces during mixing, thus increasing energy requirements [10]. Over the past decade, the functionalization of polymers by introducing chain-end functional groups or groups along the polymer backbone has increasingly gained interest because of improvements in the dynamic mechanical properties of the compounds [10,11,12]. These properties are significantly influenced by temperature variations. The glass transition temperature (*T_g_*) of the rubber matrix plays a significant role in the viscoelastic behavior of these materials. It has been observed that the loss factor *tan δ_Max_* at *T_g_* decreases with an increasing filler loading due to a decrement in the damping characteristics upon the addition of rigid filler particles [13]. Understanding this temperature-dependent behavior is crucial for predicting the performances of rubber materials under different operating conditions. Temperature variations significantly alter viscoelastic properties, affecting the responses of these rubber compounds [14,15,16].

The extent of the filler loading in rubber compounds greatly influences their rheological, mechanical, and viscoelastic properties. With an increasing filler loading, different interactions (filler-filler and filler-polymer) contribute to these properties differently [17,18,19]. Thus, understanding and optimizing the properties of these composites is essential to ensure that they meet the specific requirements of their final application. This involves not only tailoring the polymers’ properties, but also evaluating how these composite materials perform under real-world conditions, such as mechanical stress, temperature variations, and environmental exposure.

Several researchers have conducted investigations into the influences of increases in silica loading on the properties of rubber compounds. Sridharan et. al investigated the effect of a HD-silica-filled SSBR/BR blend on static and dynamic mechanical properties. They reported sharp increases in tensile moduli with an increasing silica loading up to a 50 phr filler loading, which then remained constant. The elongation at break increased with an increasing filler loading, and then decreased due to an increased stiffness and crosslink density. Also, the hardness of the compounds increased consistently with an increasing filler loading due to the increased filler network in the compound [20].

Choi et al. extensively researched the influence of filler type and content on the reinforcement properties of SBR compounds. They reported a decrease in the cure rate as the silica loading increased due to the adsorption of accelerators on the silica surface. The Mooney viscosity and mechanical properties such as hardness, modulus, and tensile strength increased upon increasing the filler content, explained by increases in filler-filler and filler-polymer interactions [6].

In the present work, the effects of increasing the silica loading in rubber compounds based on different types of (functionalized and non-functionalized) solution styrene-butadiene rubbers (SSBRs) and one type of butadiene rubber (BR) on their composite properties are described in detail. Their rheological, mechanical, and viscoelastic properties are investigated. The presented results contribute to (i) a better understanding of the changes in properties with an increasing silica loading in rubbers with varying microstructures and properties, (ii) the measurement of the percolation threshold in silica-filled compounds, which has been defined for carbon black-filled compounds in the literature [21,22,23], and (iii) the quantification of the contributions of the filler network to the viscoelastic properties.

## 2. Materials and Methods

### 2.1. Compound Formulation and Mixing Procedure

#### 2.1.1. Raw Materials

In this work, three types of SSBRs from Synthos Schkopau GmbH and one type of BR from Arlanxeo are used. Table 1 summarizes the properties of these polymers. The functionalized rubbers can chemically interact with the silica and improve their mechanical and viscoelastic properties.

The compound formulation is depicted in Table 2. To reinforce the compounds, precipitated silica (ULTRASIL^®^ 7000 GR from Evonik Industries, Wesseling, Germany), which is a highly dispersible grade of silica with a specific surface area (CTAB) of 160 m^2^/g, was used. The silane bis(triethoxysilylpropyl)disulfide TESPD (Evonik Industries, Rheinfelden, Germany) was used as coupling agent. Other ingredients, such as Treated Distillate Aromatic Extracted oil (Vivatec 500 from Hansen & Rosenthal KG, Hamburg, Germany), N-(1,3-Dimethylbutyl)-N′-phenyl-p-phenylenediamine (6PPD) Vulkanox 4020/LG from Lanxess Deutschland GmbH, Cologne, Germany, paraffin wax (Protektor G 3108 from Paramelt B.V., Heerhugowaard, The Netherlands), carbon black N330 from Apollo Tyres B.V., Enschede, The Netherlands, and a vulcanization system comprising the activators stearic acid (Edenor ST1 GS from Emery Oleochemicals GmbH, Düsseldorf, Germany) and zinc oxide (ZnO RS RAL 844 C from Arnsperger Chemikalien GmbH, Cologne, Germany), as well as a curing system comprising α-Sulphur from Eastman Chemical Corporation, Langenfeld, Germany, and N-tert-butyl-benzothiazole sulfonamide (TBBS) (Rhenogran TBBS-80) from Lanxess Deutschland GmbH, Cologne, Germany, as the primary accelerators and N,N′-Diphenylguanidine (DPG) (Rhenogran DPG) from Lanxess Deutschland GmbH, Cologne, Germany, and tetrabenzyl thiuram disulfide (Richon TBZTD OP from Richon Chem, Dalian, China) as the secondary accelerators, were also used [24,25,26].

As seen in the table above, the silane amount was adjusted according to the following formula [27]:

TESPD (phr) = 4.7 × 10^−4^ × (CTAB)_silica_ × (phr)_silica_

Similarly, the amount of TDAE was adjusted according to the following formula:

TDAE (phr) = 0.3125 × (phr)_silica_

The following Table 3 lists the variable loadings of silica (in phr and vol. %) used in the formulation, along with the adjusted amounts of silane and TDAE oil.

#### 2.1.2. Mixing Process

Table 4 shows the mixing procedure and the mixing conditions.

The ingredients were mixed in a three-stage mixing process in a 390 mL Brabender Plasticorder 350 S internal mixer (Brabender GmbH & Co. KG, Duisburg, Germany) with tangential rotors (N-rotor geometry), followed by sheeting on a two-roll mill. The rubber sheets were stored for 24 h before the subsequent mixing stages, and the fill factor was decreased by 3% after each stage to account for material loss. The additional second mixing stage was implemented to improve the extent of the silanization reaction and ensure an effective mixing process. DPG was added as an accelerator in the second stage to additionally shield the silica surface and reduce the potential absorption of the main accelerators, TBBS and TBzTD, in the final mixing stage.

#### 2.1.3. Vulcanization

The vulcanization behavior of the compounds was measured at a 0.5° (or 7.2%) strain and a frequency of 1.67 Hz at 160 °C for 30 min in the Rubber Process Analyzer (RPA) from TA Instruments (New Castle, DE, USA). Samples for tensile (sheets—95 mm × 95 mm × 2 mm) and hardness (cylinders—thickness of 12.5 mm and diameter of 30 mm) tests were prepared in the Wickert WLP 1600 (Landau, Germany) hydraulic press at 100 bar and 160 °C. The cure time for the tensile samples was 30 min and for the hardness samples was 30 + 5 min to ensure the adequate curing of the whole sample. Silica-filled compounds often show marching modulus behavior due to the reaction of unreacted silane during the mixing process, which leads to possible coupling reactions of silica–silane–polymer during vulcanization. The accelerator TBzTD suppresses this behavior to a certain extent, therefore, it was added in a small amount. The cure time was kept the same for all samples for a fair comparison [24,25,26].

### 2.2. Compound Properties

In this context, the term ‘compound properties’ refers to the characterization of raw compounds, while ‘composites’ refers to vulcanized materials containing fillers. The test methods to evaluate their rheological, mechanical, and viscoelastic properties are described as follows:

#### 2.2.1. Mooney Viscosity

The Mooney viscosity of the rubber samples was measured in the Mooney Viscometer 2000 from Alpha Technologies. This measurement was performed by the Mooney ML1 + 4 program at a temperature of 100 °C according to the ISO 289 standard [28].

#### 2.2.2. Uncured Payne Effect

Although Payne [29] described a strain-dependent reduction in the modulus of filled rubber composites for carbon-black-filled vulcanizates, is it common practice in the rubber industry to use the RPA-measured Δ*G*′ of raw mixtures as a quality parameter.

The uncured Payne effect, associated with the difference between the shear storage modulus at low and high strains (Δ*G*′*_P_*), was measured for the unvulcanized samples using the same RPA as that described in Section 2.1.3. It was measured in two sweeps to overcome the re-aggregation effect of the silica clusters during storage [30]. The test was performed from a 0.5% to 100% strain at 1 Hz and 80 °C, and the second sweep, which was performed to overcome the re-aggregation effect from the first sweep, was measured directly after the first sweep and reported.

#### 2.2.3. Shore a Hardness

The Shore A hardness of the vulcanized rubber composites was measured according to the ISO 868 standard [31]. For the test, cylinders with a thickness of 12.5 mm and a diameter of 30 mm were used. The hardness was measured four times, and the average value was used for the evaluation [32].

#### 2.2.4. Stress-Strain Behavior

The tensile test of the rubber composites was carried out on a universal testing machine (UTM) and provided direct information about the mechanical properties of the samples. The tensile properties of the vulcanized samples were measured by using a Zwick tensile tester (Model Z1.0/TH1S, ZwickRoell, Kennesaw, GA, USA) according to ISO 37. The specimens were cut into a dumbbell shape [33] from approximately 2 mm thick vulcanized sheets, and the tests were carried out at a crosshead speed of 500 mm/min with a pre-tension of 0.1 N. An average of five dumbbells was used for the analysis, and the mechanical properties discussed are the tensile strength and elongation at break [34].

#### 2.2.5. Dynamic Shear Measurements

Strain sweep measurements for vulcanized samples were carried out at 0, 25, and 60 °C on an MCR502 TwinDrive rheometer (Anton Paar Germany GmbH, Ostfildern, Germany). Dynamic shear measurements, where sinusoidal shear deformations with a variable strain amplitude were applied to the sample, were performed at three different temperatures (0, 25, and 60 °C). Stripes with a size of about 20 × 8 × 2 mm^3^ were used. During the measurements, the shear strain amplitude γ varied between 0.001% and 40%, and a frequency ω of 10 rad s^−1^ was used. The Kraus equation [35,36] was applied to evaluate the shear storage modulus *G*′*_γ_* depending on the strain amplitude.

(1) $${G^{\prime }}_{\gamma }=\frac{{G^{\prime }}_{0\;}-{G^{\prime }}_{\infty }}{1+{\left(\frac{\gamma }{\gamma_{c}}\right)}^{2m}}+{G^{\prime }}_{\infty }$$

This equation describes the sigmoidal decrease in the storage modulus as a function of the amplitude ${G^{\prime }}_{\gamma }$, with ${G^{\prime }}_{0\;}$ and ${G^{\prime }}_{\infty }$ being parameters describing the storage modulus for very small (γ → 0) and very large amplitudes (γ →∞). $\Delta G^{\prime }$ = ${G^{\prime }}_{0\;}$ − ${G^{\prime }}_{\infty }$ is a measure of the load-bearing capacity of the filler network. This is the ability of the filler network within a material to store energy and depends on how well the filler is distributed in the polymer matrix [36]. The parameter $\gamma_{c}$ characterizes the critical amplitude of the sigmoidal decay. The exponent m describes the shape of the decay, and was fixed to 0.6 for all fits performed in our study, in accordance with the experimental data.

A modified Kraus equation was used to approximate the shear loss modulus *G*″*_g_* according to Nagaraja et al. [37].

(2) $${G^{\prime \prime }}_{\gamma }=\frac{{G^{\prime \prime }}_{0\;}–\;{G^{\prime \prime }}_{\infty }}{1+{\left(\frac{\gamma }{\gamma_{c}}\right)}^{2m}}+\frac{2\left({G^{\prime \prime }}_{m\;}–\;{G^{\prime \prime }}_{\infty }\right){\left(\frac{\gamma }{\gamma_{c}}\right)}^{m}}{1+{\left(\frac{\gamma }{\gamma_{c}}\right)}^{2m}}+\;{G^{\prime \prime }}_{\infty }={{\tilde{G}}^{\prime \prime }}_{\gamma ,\;D}+{{\tilde{G}}^{\prime \prime }}_{\gamma ,F}+{G^{\prime \prime }}_{\infty }$$

The parameters ${G^{\prime \prime }}_{0\;}$ and ${G^{\prime \prime }}_{\infty }$ are the loss moduli for very small (γ → 0) and very large amplitudes (γ →∞), respectively. The parameter ${G^{\prime \prime }}_{m\;}$ quantifies the height of the peak in the loss modulus versus strain amplitude curve. The modified Kraus Equation (2) can be written as a sum of the three contributions of ${\tilde{G}\prime \prime }_{\gamma ,\;D}$, ${{\tilde{G}}^{\prime \prime }}_{\gamma ,F}$, and ${G^{\prime \prime }}_{\infty }$. According to Nagaraja et al. [37], the contribution of ${{\tilde{G}}^{\prime \prime }}_{\gamma ,F}$, which is identical to the term in the original Kraus model [35,36], represents the heat released through the breakage of the filler network, while ${{\tilde{G}}^{\prime \prime }}_{\gamma ,\;D}$ characterizes the energy dissipation due to the oscillatory deformation of the intact filler network, and ${G^{\prime \prime }}_{\infty }\;$contains all contributions to the energy dissipation that are not related to the filler network. Hence, $\Delta {G^{\prime \prime }}_{D}$ = ${G^{\prime \prime }}_{0\;}–\;{G^{\prime \prime }}_{\infty }$ and $\Delta {G^{\prime \prime }}_{F}$ = ${G^{\prime \prime }}_{m}–\;{G^{\prime \prime }}_{\infty }$ are measures of the dissipative contributions of the intact filler network and those due to the breakage of the filler network, respectively. Furthermore, it is assumed that there is no significant Payne effect in *G*′ and no peak in *G*″ for filler fractions below the percolation threshold, and that the filler network contains always glassy rubber bridges [23,37]. 

## 3. Results and Discussion

### 3.1. Mooney Viscosity

**Figure 1:**
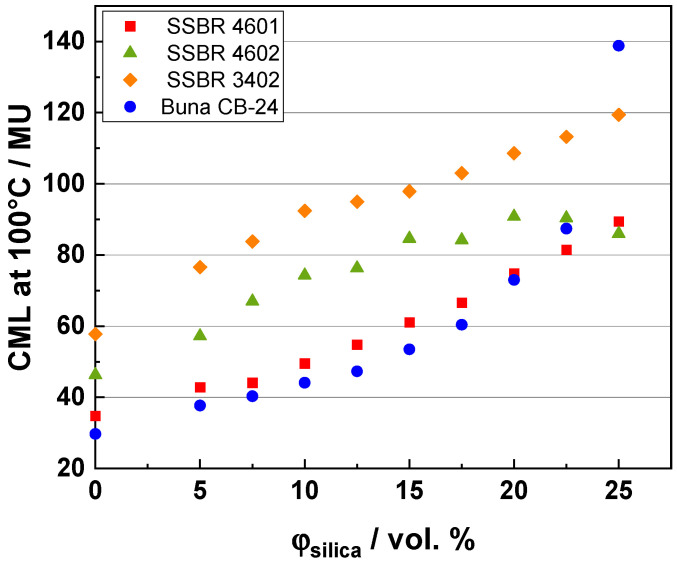
Mooney viscosities of different compound systems.

Mooney viscosity forecasts the processability of a compound and is a key parameter in the rubber industry for quality control and process optimization [38]. It helps to ensure that rubber compounds have the appropriate flow characteristics for their intended applications. Figure 1 shows the Mooney viscosities of different compound systems with varying silica loadings. CML represents the compound Mooney viscosity measured with a large rotor. With an increasing filler loading, the expectation is that the Mooney viscosity would increase due to the increasing stiffness of the compound.

As can be seen from Figure 1, the viscosity initially increases linearly for the four compound systems due to an increase in the proportion of hard filler particles in the polymer matrix. For the unfilled compounds, the viscosity follows the order of Buna CB24 < SSBR 4601 < SSBR 4602 < SSBR 3402, which is in agreement with the intrinsic viscosities of the polymers (see Table 1). For the Buna CB24 compounds, the viscosity increases linearly until reaching a 15 vol. % silica loading, and then increases drastically, which would be expected from a rheological standpoint according the Krieger-Dougherty equation [39], thus indicating the inability of BR to incorporate [40] higher loadings of silica and worsening its processability. This has been well-defined in the literature, as follows: BR has a lower compatibility with silica as compared to SBR because of its highly linear structure and absence of aromatic groups, thus resulting in a small-bound rubber fraction or weaker filler-polymer interaction [41,42]. For all compounds, the viscosity increases linearly from the unfilled compound to the highest-filled compound, with the lowest values observed for the Buna CB24 and SSBR 4601 composites and higher values for SSBR 4602 and SSBR 3402. The functional groups can interact with the silica via silane [43] or directly with silica, thus reducing the filler–filler interaction and, hence, reducing the viscosity of the compound. Here, a further contribution comes from the adjusted silane and TDAE content with a rising silica amount. The viscosities for compounds with SSBR 3402 increase linearly and are the highest as compared to other systems because of the highest intrinsic viscosity of SSBR 3402 among all the polymers. The lower styrene and vinyl content can result in a relatively lower compatibility with silica as compared to SSBR 4601 and SSBR 4602 and, hence, could result in greater filler-filler interactions, thus increasing the compound viscosities.

### 3.2. Uncured Payne Effect

**Figure 2:**
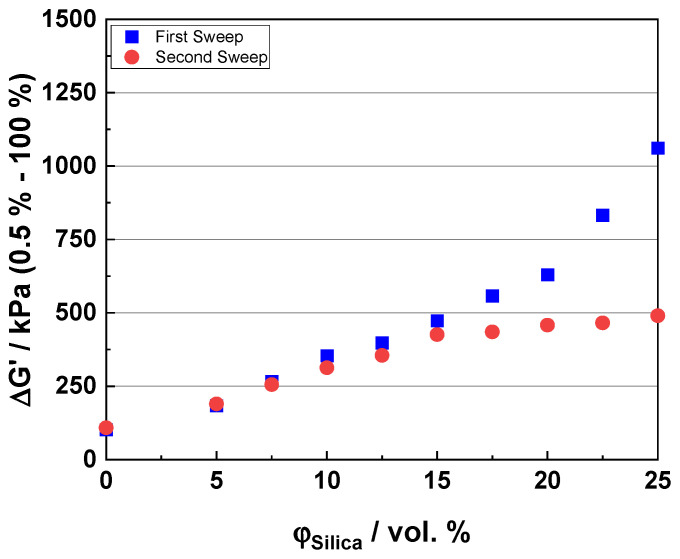
Uncured Payne effects for SSBR 4602 compounds. The percolation threshold is observed between 15 and 17.5 vol. % filler loading.

The uncured Payne effect is commonly understood as a measure of the extent of the filler-filler interaction in the raw compound. The higher the measured value, the higher the interaction [30]. The Payne effect values increase with an increasing filler loading due to the formation of the filler network. As mentioned in Section 2.2.2, this measurement is conducted in two sweeps to overcome the re-aggregation effect of the silica clusters during storage. The percolation threshold, which indicates the formation of a full filler network, is found by observing the differences between the two strain sweeps. The point at which a percolating filler network (infinite cluster) forms with an increasing filler content is referred to as the percolation threshold [44]. Figure 2 shows the two sweeps from the uncured Payne effect measurement for the SSBR 4602 compounds. The Δ*G′_P_* on the Y-axis is the difference between the storage modulus at a low (0.5%) and high (100%) strain, and *Φ_silica_* on the X-axis is the silica loading in vol. %. It is seen that the difference in the measurements starts appearing between a 15 and 17.5 vol. % filler loading, which indicates the percolation threshold point. This implies that, below a 15 vol. % loading, after the first sweep of the measurement, the filler network is not yet formed, due to an insufficient amount of filler in the compound [21,22]. This can be seen by the overlap of the first and second sweep points up to around a 15 vol. % loading. After this loading, the differences in the two sweeps are observed. This indicates that the amount of filler is sufficient to form a filler network (or an infinite solid cluster going through the whole raw compound), and that the filler loading is above the percolation threshold. This is consistent with all the compound systems.

The filler network comprising soft clusters is destroyed after the first sweep, and the network comprising hard clusters, which are reversibly formed, gives the true extent of the Payne effect, which has been described for vulcanized systems [45].

**Figure 3:**
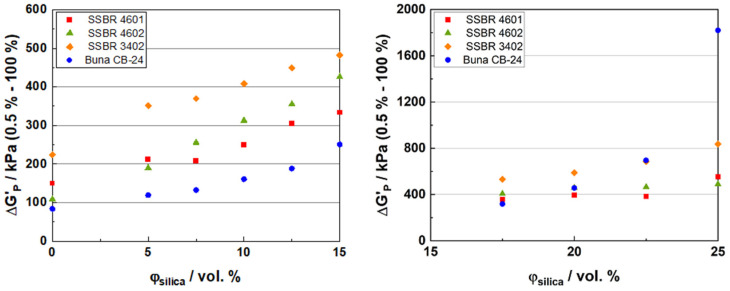
Uncured Payne effect: (**left**) below the percolation threshold and (**right**) above the percolation threshold for the raw compound systems.

Figure 3 shows the uncured Payne effect (second sweep) at 80 °C for the final-stage compounds of the four compound systems. The Δ*G′_P_* on the Y-axis is the difference between the storage modulus at a low (0.5%) and high (100%) strain, and *Φ_silica_* on the X-axis is the silica loading in vol. %. The left graph shows the results for the compounds below the percolation threshold and the right graph shows the results for above the percolation threshold.

In Figure 3 (left), compounds with Buna CB24 and SSBR 3402 show the lowest and highest Payne effect, respectively. At low filler loadings (below the percolation threshold), the Payne effect follows the order of Buna CB24 < SSBR 4601 ≈ SSBR 4602 < SSBR 3402. This can be related to the storage modulus (*G′_P_*) values of the compounds at low strains. The *G′_P_* depends on the rheological properties of the compound (viscosity, filler-filler, and filler-polymer interactions, etc.). At low filler loadings, the filler-filler interaction is less pronounced and the polymer phase dominates, and hence, due to their lower compound viscosities, the CB24 compounds have a lower uncured Payne effect. Among the SSBRs, the SSBR 3402-containing compounds show the highest uncured Payne effect. This is because these SSBR 3402 compounds have lower styrene and vinyl content as compared to the other two SSBRs, and hence, will have a lower compatibility with silica, thus resulting in more filler–filler interactions. This is also the reason for their highest Mooney viscosities among the SSBRs. Also, similar values are observed for SSBR 4601 and SSBR 4602. Although a lower uncured Payne effect is expected from a functionalized polymer such as SSBR 4602 because of the additional interaction between the functional groups of the polymer and a polar filler such as silica, it has been found for other polymer systems that the reaction of functional groups with the silica predominantly takes place via silane [43]. However, it also depends on the type of end-chain functionalization, the position of the functionalization, and the accessibility of the functionalization. Another possible explanation can be that the dump temperatures for SSBR 4602 compounds were kept lower than those for SSBR 4601 to avoid polymer degradation, and hence, a lower degree of silanization can explain this behavior. In Figure 3 (right), above the percolation threshold, as the filler loading increases, compounds with CB24 show a much higher uncured Payne effect due to the incompatibility of BR with silica, as observed in the Mooney viscosity results [41,42]. This can be explained by the Hansen Solubility Parameters for polymers and fillers. The values for SBR (18.9 MPa^1/2^) and silica surface (19.4 MPa^1/2^) are closer to each other as compared to the values for BR (16.9 MPa^1/2^) and silica surface (19.4 MPa^1/2^). This indicates that SBR has a better compatibility with silica as compared to BR [46]. Uncured Payne effect measurements were also used as indication of the extent of the micro-dispersion in the compounds [47]. Additionally, due to the combination of its low compatibility with silica and its highly linear structure, the micro-dispersion in BR is expected to be poorer as compared to SBR, as the filler is more likely to occupy the space between the bulky benzyl groups in SBR, thus improving the micro-dispersion [48]. SSBR 3402 compounds again have the highest uncured Payne effect values among the SSBRs which can be related to the Mooney viscosity results. As compared to SSBR 4601 and SSBR 4602, SSBR 3402 has a lower styrene and vinyl content (and a higher linear structure), and hence, its compatibility with silica reduces. This results in a higher uncured Payne effect in SSBR 3402 as compared to SSBR 4601 and SSBR 4602. The differences between SSBR 4601 and SSBR 4602 are negligible, which has a similar explanation as above. For the highest-filled compounds, the Payne effect follows the following order: SSBR 4602 ≈ SSBR 4601 < SSBR 3402 < Buna CB24. Though CB24 has the lowest polymer viscosity, it has the highest Payne effect for the highest-filled compound because of its lower compatibility, as explained by the Hansen Solubility Parameter earlier, compared to the SSBR compounds. The highest-filled CB24 compound also has the highest Mooney viscosity because of its lower compatibility with silica, thus resulting in a stronger filler–filler network. As the filler loading increases, the shear forces in the compound also increase. This usually results in a better filler dispersion. Among the SSBRs, 3402 shows the highest uncured Payne effect. This is because of the lower styrene and vinyl content in SSBR 3402 as compared to SSBR 4601 and SSBR 4602, so the uncured Payne effect is higher for SSBR 3402 compounds with increasing filler loadings as compared to the other two SSBRs [49].

Recently, it was found that a stronger Payne effect directly correlates with the higher connectivity of filler networks, rather than the higher homogeneity of nanofillers [50].

### 3.3. Cure Behavior

**Figure 4:**
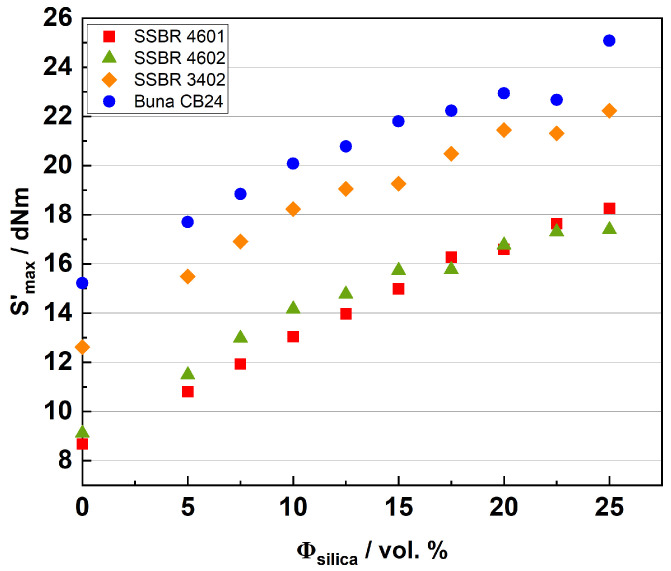
Cure characteristics (*S*’*_max_*) for compound systems.

The cure characteristics of the compounds can be explained by analyzing *S*’*_max_*, which is the maximum torque (*S*’*_max_*) attained during curing. Figure 4 shows the results for *S*’*_max_* with varying silica loadings for the compound systems.

The *S*’*_max_* follows the order of Buna CB24 > SSBR 3402 > SSBR 4601 ≈ SSBR 4602 for the unfilled compounds. This is in accordance with the micro-structures of the polymers. A higher degree of unsaturation in CB24 results in a higher extent of crosslinking, thus resulting in a higher *S*’*_max_* value among the unfilled compounds [51]. Similarly, SSBR 3402 has a higher BR content (or a lower styrene and vinyl content) as compared to SSBR 4601 and SSBR 4602. As the filler loading increases, the overall trend remains the same for all the compound systems, as follows: an increase in *S*’*_max_* is observed. This is because of increases in the filler–polymer and filler–filler interactions and their contributions to the cure torque as the filler loading increases [52]. For low silica contents up to 17.5 vol.%, SSBR 4602 shows higher *S*’*_max_* values than SSBR 4601, caused by the contribution of the functionalization of SSBR 4602. With a rising silica content, other aspects like filler-filler and filler-polymer interactions become predominant. Depending on the polymer systems and their modification, the reaction of the functional groups with silica takes place more or less through the silane. Also, the number of end-chain functional groups is much lower as compared to the lengths of polymer chains, and hence, the effects on the cure results are hardly visible due to the lower frequency of functional groups. This was observed by Yamada et al. [43]. With increasing filler loadings, SSBR 3402 compounds show higher *S*’*_max_* values than the other two SSBRs because of a higher BR content (or a lower styrene and vinyl content), leading to more possibilities for crosslinking reactions. At very high filler loadings, some deviations from the trend are observed.

### 3.4. Shore a Hardness

**Figure 5:**
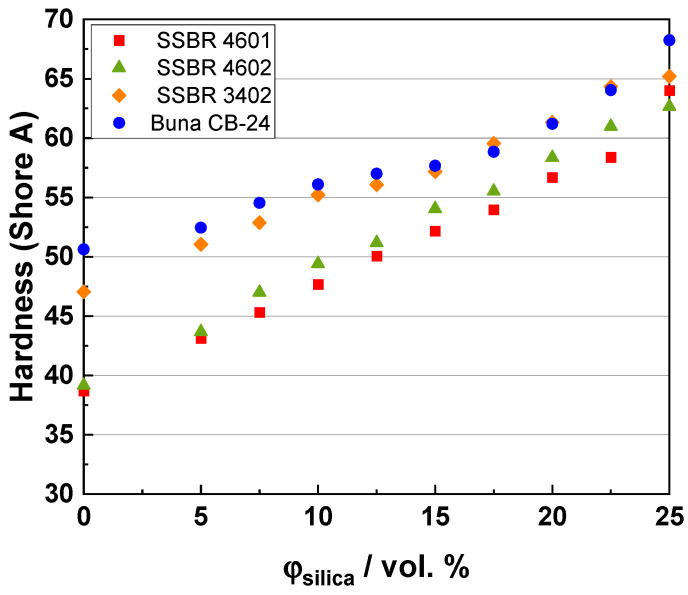
Hardness for composite systems.

Figure 5 shows the hardness results for the composite systems. The hardness of the composites majorly depends on the crosslink density and on the contribution from the filler-filler interaction. This has been well described in the literature [53].

The hardness increases with an increasing filler content because of an increasing proportion of rigid filler particles in the composites. For the unfilled samples, the hardness follows the following order: SSBR 4601 < SSBR 4602 << SSBR 3402 << Buna CB24. The degree of crosslinking is the highest in the Buna CB24 compounds [54], as discussed in the previous section, and hence, they have the highest hardness, followed by the SSBR 3402 compounds. As the filler loading increases, the differences in hardness for these two mixtures decrease (except for the last compounds). This can be explained by the fact that hardness is a quasi-static measurement and the filler content dominates over the crosslink density of the compounds, especially at high loadings. Also, the macro- and micro-dispersion of filler majorly affects the hardness of these compounds at high filler loadings. For the last compound with Buna CB24, the incompatibility of silica with BR leads to high filler–filler interaction, thus increasing the hardness. SSBR 4602 compounds show slightly higher hardness values with an increasing filler content as compared to SSBR 4601, except for the highest-filled compound. This could be because of the additional crosslinks formed because of the reaction between functional groups and silica, resulting in a greater filler-polymer interaction, and hence, a higher hardness. Another reason could be the higher intrinsic viscosity of the SSBR 4602 as compared to the SSBR 4601 polymer because of functionalization, and this can directly affect the hardness of the compounds. A sudden increase in hardness for the highest-filled SSBR 4601 compound could be due to a greater filler–filler interaction as compared to the SSBR 4602 compound. This interaction dominates over the filler-polymer interaction and polymer crosslinking at very high filler loadings, and hence, can have a higher contribution to the compound hardness.

### 3.5. Stress-Strain Behavior

**Figure 6:**
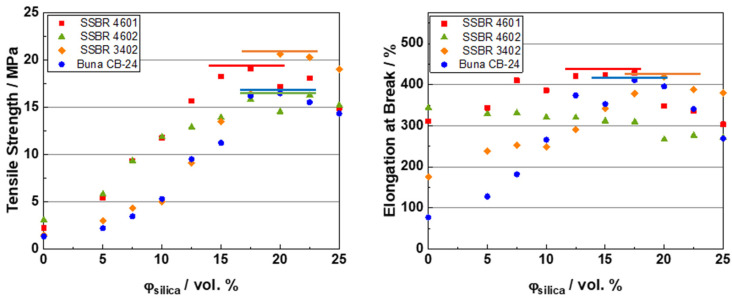
Stress-strain properties for the composite systems: (**left**) tensile strength and (**right**) elongation at break.

The stress-strain properties are affected by various factors such as the molecular weight of the polymer and filler-filler and filler-polymer interactions, etc. [42]. Figure 6 shows the tensile strength (left) and the elongation at break (right) results for the composite systems.

For all the systems, a plateau is observed in the tensile strength, as well as in the elongation at break (except for the SSBR 4602 composites), thus indicating an optimum filler loading. This has been well described in the literature [55,56]. As the filler loading increases, the reinforcement effect of the filler leads to increases in both of these properties. However, at high filler loadings, these properties start to decrease as a result of the stress amplification between filler clusters.

The tensile strength for SSBR 4602 composites near and above the percolation threshold is lower as compared to SSBR 4601 compounds due to a greater filler-filler interaction than that in SSBR 4601 compounds. Though the polymer-filler interaction is greater in SSBR 4602 compounds due to the functionalization of the polymer, the filler-filler interactions dominate at high loadings, thus resulting in a higher tensile strength for SSBR 4601 compounds. Also, below the percolation threshold, a higher styrene content (π-π interactions) may dominate and lead to a higher tensile strength in SSBR 4601 and SSBR 4602 compounds as compared to SSBR 3402 and Buna CB24 compounds. Above the percolation threshold, the filler effect dominates and the strong filler-filler interactions result in a higher tensile strength for SSBR 3402 compounds. A combination of molecular weight and filler-filler and filler-polymer interactions affects the tensile properties at high filler loadings. The high molecular weight and high filler-filler interactions in Buna CB24 composites bring their tensile strength to the same level as that of SSBR 4602 compounds, which have a lower molecular weight and lower filler-filler interactions.

The elongation at break for the unfilled samples follows the order of Buna CB24 < SSBR 3402 < SSBR 4601 < SSBR 4602. In the absence of filler, and at low filler loadings, the polymer dominates the elongation at break. As BR has a higher amount of double bonds in the main chain and, therefore, a higher expected degree of crosslinks per unit volume (vs. SBR), a high external force results in an earlier strain with higher stresses at a constant strain compared to SBR, thus reducing the elongation at break. Among the SSBRs, SSBR 3402 has a lower styrene and vinyl content, resulting in a higher amount of double bonds in the main chain and, therefore, a higher degree of crosslinks as compared to unfilled SSBR 4601 and SSBR 4602 samples. This results as well in the lowest elongation at break. The unfilled SSBR 4601 and SSBR 4602 samples show similar values, having a similar amount of double bonds in their main chain. Additionally, the highly linear structure of BR results in a high packing efficiency of polymer chains per unit volume, whereas SBR has bulky aromatic groups, which lead to a lower packing efficiency and more entanglements, resulting in a higher polymer mobility [57]. Among the SSBRs, SSBR 4602 has the highest elongation at break because the functional groups present at the chain ends can interact with themselves, thus extending the chain lengths and the elongation at break. This is followed by SSBR 4601, which is non-functionalized for silica, and SSBR 3402, which has a lower styrene and vinyl content, thus resulting in a relatively higher packing efficiency and, subsequently, a lower elongation at break. As the filler loading increases, a plateau is observed in the elongation at break results, indicating an optimum filler loading for all compound systems except SSBR 4602. The elongation at break can also be affected by the mixing process and the ingredients. The different ratio of chain scission in SSBR 4602 and SSBR 3402 and the type of interaction of oil with both the polymers could play roles in determining the extent of elongation of the compounds. The trend in these results can be well supported by the findings from Sridharan et al. [20]. Though the SSBR 4601 and SSBR 4602 composites have a similar hardness, the elongation at break for SSBR 4602 samples appears to follow an unusual trend with an increasing filler loading, as follows: it is highest for the unfilled compound and slightly decreases as the filler loading increases. This is because the contributions from the filler and polymer can have varying effects on the mechanical properties. For example, greater filler-filler and lower filler-polymer interactions in the SSBR 4601 compounds and lower filler-filler and greater filler-polymer interactions in SSBR 4602 compounds can result in similar hardness values. However, the influences of filler-filler and filler-polymer interactions change as the filler loading increases. At higher loadings, the molecular weight of the polymer and the filler network dominate, and hence, SSBR 3402 has the highest elongation at break.

### 3.6. Dynamic Shear Measurements

**Figure 7:**
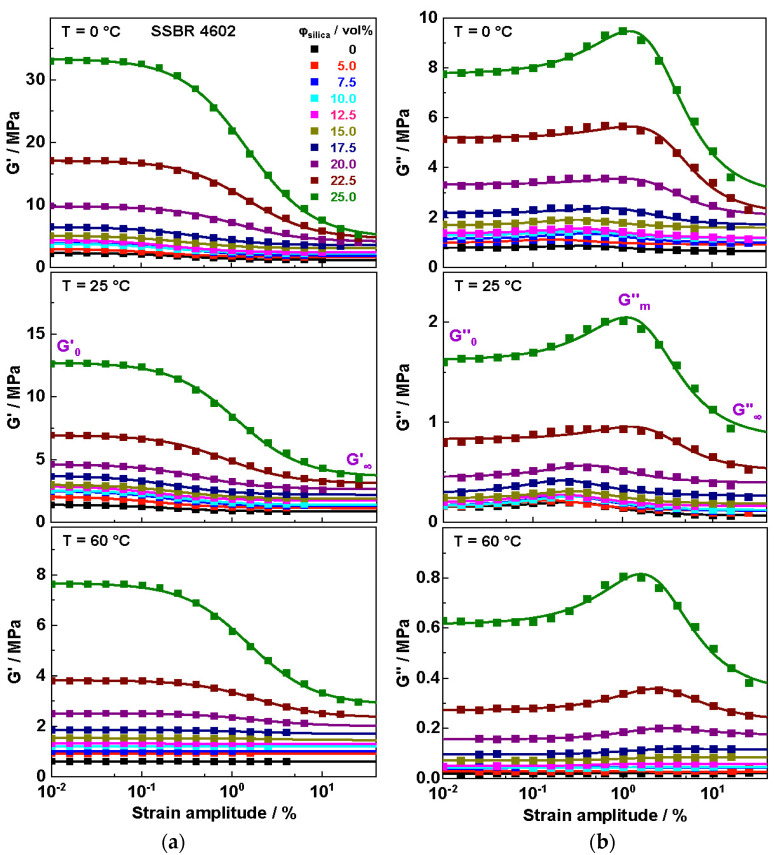
Strain-dependent (**a**) shear storage modulus *G*′ and (**b**) shear loss modulus *G*″ data for SSBR 4602 composites containing different amounts of silica measured at different temperatures. The lines in parts (**a**) and (**b**) are fits based on Equations (1) and (2), respectively.

The influences of temperature and filler content on the reinforcement and energy dissipation properties of the filler network in different SSBR and BR compounds are investigated by using strain sweep measurements of vulcanized samples. Data for SSBR 4602 compounds with different silica contents are shown in Figure 7 as a representative example.

Sudden increases in the storage modulus ${G^{\prime }}_{0\;}$ and loss modulus ${G^{\prime \prime }}_{0\;}$ at small strain amplitudes (<0.1%) are observed above a certain filler content. This effect can be traced back to the formation of a filler network in highly filled rubber systems connected with a pronounced Payne effect Δ*G*′ in data for the shear storage modulus depending on the strain amplitude. The rises in ${G^{\prime }}_{0\;}$and ΔG’ depending on the silica content start close to the percolation threshold obtained in RPA measurements on uncured compounds at 15–17.5 vol. %. Slight differences based on the characterization techniques used are definitively possible, since the frequency for Payne effect measurements is 1 Hz for RPA and 10 rad s^−1^ for dynamic shear measurements. Note that the first sweeps for the vulcanized composites are reported in this section, where the filler network is basically intact at the beginning of the strain sweeps. 

The sigmoidal change in G′*_g_* from low to high strain amplitudes by ΔG′ is well in accordance with Equation (1) and basically reflects the load-bearing capacity of the filler network for compounds with high filler contents Φ > Φ_c_ (cf. Figure 7). Here, the decrease in G’*_g_* with increasing strain amplitudes indicates the breakage of the filler network commonly discussed as the origin of the Payne effect [30]. In parallel, a peak in G″*_g_* is detected in strain sweeps for all samples with silica filler contents above the percolation threshold *F_c_*. In addition, an underlying sigmoidal decrease in G″ is observed in all investigated rubber composites, as reported earlier for related composites [23,37]. According to Kraus, the formation of the peak in G″*_g_* depending on the strain amplitude is related to the heat released when bridges in the filler network break [35,37]. More recently, the relevant contributions of ΔG″*_g,F_* and ΔG″*_g,D_* have been associated with the heat produced during the fracturing of glassy rubber bridges and dissipation due to an oscillatory deformation of intact glassy rubber bridges being part of the filler network, respectively. The values of $\Delta {G^{\prime \prime }}_{F}$ and $\Delta {G^{\prime \prime }}_{D}$, quantifying peak and step heights, respectively, can be taken from fittings according to Equation (2), commonly approximating the experimental data quite well. Obviously, both parameters $\Delta {G^{\prime \prime }}_{F}$ and $\Delta {G^{\prime \prime }}_{D}$ quantify different contributions of the filler network to dissipation. 

**Figure 8:**
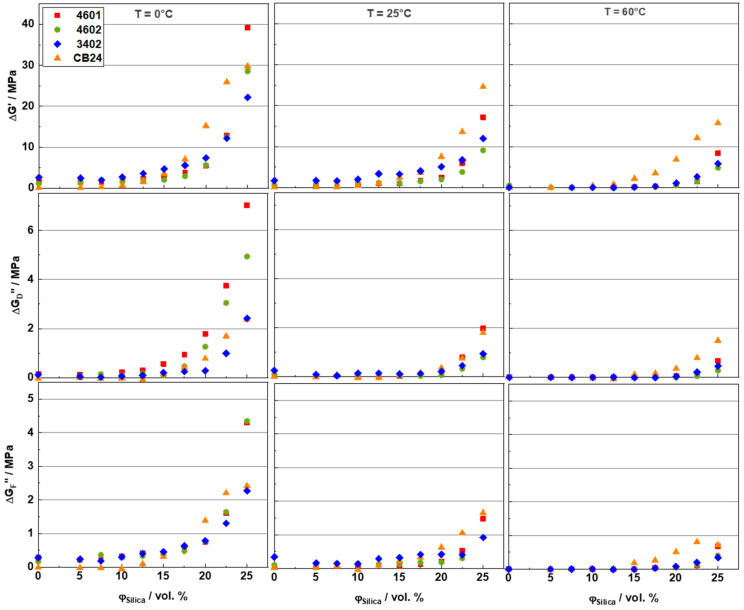
Fit parameters for SSBR and BR compounds with different silica contents from strain sweeps measured at 0, 25, and 60 °C.

Figure 8 shows the fit parameters from Equations (1) and (2)—$\Delta G^{\prime }$, $\Delta {G^{\prime \prime }}_{F}$, and $\Delta {G^{\prime \prime }}_{D}$—for all SSBR and BR compounds investigated in this work. Considering the $\Delta G^{\prime }$ values at 25 °C, a weak influence of the rubber matrix on the percolation threshold *F_c_* is indicated. Only the *F_c_* values for BR are seemingly about 2.5 vol. % lower than those for the SSBR samples. This might be related to differences in the interaction between the filler and the rubber. As explained earlier, the lower compatibility of BR with silica can influence the filler dispersion and, thus, also cause a slight shift of the percolation threshold *F_c_*. Also, no significant dependence of the percolation threshold on temperature is observed, as reported earlier for other rubber compounds [33]. Comparing the tiny $\Delta G^{\prime }$ values for rubber composites with low silica contents (*F* << *F_c_*), one observes near-room-temperature trends similar to those found in RPA measurements on uncured samples. The $\Delta G^{\prime }\;$values are highest for SSBR 3402 and lowest for the BR matrices. The related $\Delta G^{\prime }$ values for SSBR 4601 and SSBR 4602 are in between. Above the percolation threshold (*F* >> *F_c_*), a sudden increase in $\Delta G^{\prime }$ is observed for all rubber matrices with an increasing filler content, which corresponds to a higher load-bearing capacity of the filler network. Interestingly, BR shows significantly higher $\Delta G^{\prime }$ values compared to the SSBR compounds for such high silica contents, in contrast to the behavior obtained for very low silica contents. Most likely, this effect is related to the poor compatibility of BR with silica, resulting in a strong filler network [58]. From the experimental findings, it can be concluded that the load-bearing capacity of the filler network depends not only on the filler content and temperature, but also on the type of rubber.

Further, it can be seen in Figure 8 that the strength of the filler network $\Delta G^{\prime }$, as well as the dissipative contributions $\Delta {G^{\prime \prime }}_{D}$ and $\Delta {G^{\prime \prime }}_{F}$, related to the filler network in highly filled compounds (*F* > *F_c_*), decrease significantly with an increasing temperature. Considering the dissipative contributions $\Delta {G^{\prime \prime }}_{D}$ and ${{\Delta G}^{\prime \prime }}_{F}$ in more detail, one can conclude from Figure 8 that their dependencies on filler content and temperature are qualitatively similar to those of $\Delta G^{\prime }$. From their values in Figure 8, specific composite differences in $\Delta G^{\prime }$ are also confirmed. In particular, Buna CB24 compounds commonly show the highest energy dissipation, except $\Delta {G^{\prime \prime }}_{D}\;$at 0 °C, where the SSBR samples 4602 and 4601 approach their *T_g_.* This finding is probably related to the fact that the filler network in BR is really strong, resulting in a filler network with many bridges in Buna CB24 composites.

The systematic dependences of $\Delta G^{\prime }$, $\Delta {G^{\prime \prime }}_{F}$, and $\Delta {G^{\prime \prime }}_{D}$ on temperature, filler content, rubber type, and frequency have recently been explained by the existence of glassy rubber bridges being part of the filler network [23,37,59,60] in certain contradiction to the original picture, where the reinforcement contributions of the filler network are associated solely with filler-filler interactions [17,18,19]. Viscoelastic glassy rubber bridges in the filler network give a suitable explanation for the fact that the ΔG′ values systematically decrease with an increasing temperature and decreasing frequency. It is assumed that the number of glassy rubber bridges decreases due to the sequential softening of glassy rubber layers with a thickness in the one nanometer range surrounding the filler particles and interconnecting neighboring nanoparticles [37]. Glassy rubber bridges are formed, which enable the formation of the filler network and are responsible for its viscoelastic properties. With an increasing temperature, the thickness of these glassy rubber layers decreases, resulting in fewer and fewer glassy rubber bridges interconnecting filler particles or clusters and a reduction in ΔG′. The temperature-frequency dependence of $\Delta G^{\prime }$ is not considered in the original picture, assuming that filler-filler interactions control the filler network. Along this line, another possible mechanism contributing to the decrease in ΔG′ might be the faster rate of desorption of the filler from the polymer surface as the temperature increases [61]. Note that, with an increasing temperature, the load-bearing capacities ΔG′ amount to nearly equal levels for the SSBRs, possibly indicating that differences in microstructure and bulk *T_g_* become less important. However, there are still higher ΔG′ values for BR compounds at 60 °C, supporting the explanation that the higher connectivity of filler is most relevant in this case.

**Figure 9:**
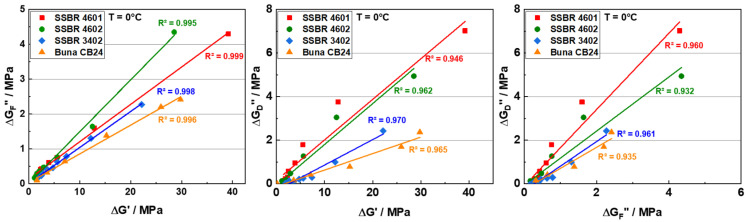
$\Delta {G^{\prime \prime }}_{F}\;vs.\;\Delta G^{\prime }$; $\Delta {G^{\prime \prime }}_{D}\;vs.\;\Delta G^{\prime }$; and $\Delta {G^{\prime \prime }}_{D}\;vs.\;\Delta {G^{\prime \prime }}_{F}$ for SSBR and BR compounds at a temperature of 0 °C.

Figure 9 shows three plots indicating correlations between the parameters $\Delta G^{\prime }$, $\Delta {G^{\prime \prime }}_{D}$, and $\Delta {G^{\prime \prime }}_{F}$ at 0 °C for all the investigated rubber composites. Each of the three parameters shows, in a reasonable approximation, a linear dependence on the other two parameters. This finding supports the idea [23,37] that the filler-network-related contributions to reinforcement ($\Delta G^{\prime }$) and dissipation ($\Delta {G^{\prime \prime }}_{D}$ and $\Delta {G^{\prime \prime }}_{F})$ depend on a similar control parameter and have the same physical origin. According to Ref. [37], this parameter might be the initial number of glassy rubber bridges in the filler network in its undeformed state. All three fit parameters should, therefore, be proportional to the number of existing glassy rubber bridges, explaining the proportionalities observed in Figure 9. In addition, the type of rubber matrix appears to have a significant effect on the number of glassy rubber bridges, since the thickness of the glassy rubber layer is dependent on the filler-rubber interaction. Commonly, this thickness decreases with an increasing temperature, resulting in the disappearance of glassy rubber bridges occurring in the region between neighboring filler particles with a distance of about 1–2 nanometers from each other. The superposition of the immobilized layers results, then, in the formation of glassy rubber bridges that connect filler particles and clusters. Due to fewer rubber bridges in the filler network, the load-bearing capacity, dissipation, and heat released related to the filler network decrease with an increasing temperature. This shows that the temperature-dependent viscoelastic properties of rubber compounds may also be controlled, to a large extent, by the number of glassy rubber bridges [23,37,59,60].

In summary, the polymer microstructure and filler loadings within it have a profound impact on the rheological and viscoelastic properties of filled rubber composites. For practical applications, such as in tire tread compounds, selecting an optimal combination of these parameters is essential to achieve an optimum balance of properties without compromising any critical aspects. For example, while the functionalization of polymers enhances both static and dynamic mechanical properties—beneficial for durability and performance—it also increases the viscosity of the composite, leading to challenges in processability.

Similarly, adjusting the filler loading can improve certain mechanical characteristics, such as wear resistance or grip, but may also alter the elasticity and energy dissipation properties, which are crucial for maintaining comfort, safety, and fuel efficiency in tire applications. Therefore, the careful tuning of both the polymer structure and filler composition is required to ensure that such compound meet the performance standards for their intended applications, balancing durability, elasticity, and ease of manufacturing without sacrificing essential performance attributes.

## 4. Conclusions

Rubber compounds with three SSBR types (SPRINTAN^®^ 4601, SPRINTAN^®^ 4602, and SPRINTAN^®^ 3402) with varying styrene and vinyl contents, molecular weights, and functionalization, and one BR type (Buna^®^ CB24) were prepared with different loadings of a highly dispersible grade of silica (ULTRASIL^®^ 7000 GR). The rheological, cure, mechanical, and viscoelastic properties of these rubber samples were analyzed. 

Overall, the Mooney viscosity and hardness of the composites increased with an increasing silica loading because of increasing filler-polymer and filler-filler interactions as the amount of filler was increased. At high filler loadings, the viscosity for Buna CB24 compounds increased drastically because of the incompatibility of BR with silica, resulting in a situation where high loadings of filler led to a higher connectivity of the filler network. A higher maximum cure torque was observed in Buna CB24 compounds because of a high cis content, which resulted in more possibilities for crosslinking due to a higher degree of unsaturation in the polymer chains. Among the SSBRs, the SSBR 3402 compounds had a higher maximum torque because of a lower styrene and vinyl content, or a higher cis content which resulted in a higher degree of crosslinking. The percolation threshold in the compounds was measured to be between a 15 and 17.5 vol. % silica loading for all the compounds. The Payne effect measured on cured rubber compounds by dynamic shear measurements gave quite similar values for the percolation threshold. Only for BR was a slightly higher percolation threshold (2.5 vol. %) indicated. The tensile strength and elongation at break increased with an increasing silica loading to a maximum and then decreased, thus indicating an optimum filler concentration. Below the percolation threshold, a higher styrene content (π-π interaction) may have dominated and led to a higher tensile strength in the SSBR 4601 and SSBR 4602 compounds as compared to the SSBR 3402 and Buna CB24 compounds. For elongation at break, a highly linear structure of BR resulted in a high packing efficiency of polymer chains per unit volume, thus resulting in lower values as compared to SSBRs. Above the percolation threshold, the filler dominated and the strong filler-filler interactions combined with a high polymer molecular mass resulted in a higher tensile strength and elongation at break for SSBR 3402 composites. Hence, a combination of molecular weight and filler-filler and filler-polymer interactions probably controlled the tensile properties at high filler loadings. The viscoelastic properties of the filler network in the cured rubber composites were quantified by strain sweeps using dynamic shear measurements. It was observed that the type of rubber, the filler content, and the temperature had significant influences on the load-bearing capacity of the filler network Δ*G*′ and the energy dissipation behavior in the loss modulus quantified by Δ*G*″_F_ and Δ*G*″*_D_*. It was demonstrated that the glassy rubber bridge model can explain the major findings in the fit parameters from the Kraus Equation (1) and the modified Kraus Equation (2). From the results, it can be concluded that the three factors of rubber type, temperature, and filler content affected the number of glassy rubber bridges in the initial undeformed state. The change in the shear moduli (storage and loss) of the compounds was not only dependent on the chemical composition of the glassy rubber bridges (filler-polymer interactions), but also on the filler network topologies related to the filler dispersion, which was further dependent on the compatibility of the polymers with the filler. These parameters can, therefore, strongly influence the rheological and viscoelastic behavior of composites.

## Figures and Tables

**Table 1:** Selection of polymers and their properties.

Polymer	SPRINTAN^®^ 4601	SPRINTAN^®^ 4602	SPRINTAN^®^ 3402	BUNA CB24
Functionalization	Yes, for carbon black	Yes, for silica	Yes, for silica	No
ML1 + 4 (100 °C)/MU	50	63	70	44
Density/g/cm^3^	0.934	0.935	0.925	0.91
Styrene/%	21	21	15	-
Vinyl/%	63	63	30	-
1,4-cis/%	-	-	-	98
Molecular Weight	Low	Low	High	High
*T_g_* (DSC)/°C	−25	−25	−62	−107

**Table 2:** Compound formulation.

Ingredients	Amount (phr)	Role
Rubber	100	Polymer
ULTRASIL^®^ 7000 GR	variable	Filler
Si 266^®^	adjusted	Silane coupling agent
Edenor ST1 GS	2	Activators
ZnO RS RAL 844 C	2	
Vulkanox 4020/LG	2	Antioxidant
Protektor G 3108	2	Antiozonant
N330	3	Antioxidant and colorant
Vivatec 500	adjusted	Lubricant
α-Sulphur	1.4	Vulcanization system
Rhenogran TBBS-80	1.5	
Rhenogran DPG	1.5	
Richon TBZTD OP	0.4	

**Table 3:** Variable loadings of silica and silane and TDAE oil content.

Silica (vol. %)	Silica (phr)	Silane (phr)	TDAE Oil (phr)
0.0	0	0	0
5.0	13	0.9	4
7.5	21	1.5	6
10.0	29	2.1	9
12.5	38	2.8	12
15.0	49	3.6	15
17.5	61	4.4	19
20.0	74	5.4	23
22.5	90	6.5	28
25.0	108	7.8	34

**Table 4:** Mixing procedure.

**1st Stage**		
Fill factor: 72% Initial temperature: 80 °C Initial rotor speed: 70 rpm	Time (mm:ss)	Workflow
	00:00	Add raw rubber, start measurement
	00:20	Ram down
	01:00	Ram up, add 1/2 or 2/3 (silica + silane)
	01:30	Ram down
	02:30	Ram up, add oil, chemicals, 1/2 or 1/3 (silica + silane)
	03:00	Ram down
	04:00	Ram up, sweep for 15 s
	04:15	Ram down, isothermal mixing at 140 °C
	07:00	Dump and check weight, dump T, sheet out on mill × 5
**2nd Stage**		
Fill factor: 69% Initial temperature: 80 °C Initial rotor speed: 80 rpm	Time	Workflow
	00:00	Add batch stage 1, ram down, start measurement
	00:50	Ram up, add DPG
	01:00	Ram down, adjust speed, isothermal mixing at 140
	05:00	Dump and check weight, dump T, sheet out on mill × 5
**3rd Stage**		
Fill factor: 66% Initial temperature: 50 °C Initial rotor speed: 50 rpm	Time	Workflow
	00:00	Add batch stage 2, ram down, start measurement
	00:30	Ram up, add vulcanization system, ram down
	03:00	Dump and check weight, dump T, sheet out on mill × 5

## Data Availability

Data are contained within the article.
